# Differences in rituximab use between pediatric rheumatologists and nephrologists for the treatment of refractory lupus nephritis and renal flare in childhood-onset SLE

**DOI:** 10.1186/s12969-021-00627-w

**Published:** 2021-08-30

**Authors:** Mileka Gilbert, Beatrice Goilav, Joyce J. Hsu, Paul J. Nietert, Esra Meidan, Annabelle Chua, Stacy P. Ardoin, Scott E. Wenderfer, Emily von Scheven, Natasha M. Ruth, for the Pediatric Nephrology and Rheumatology Collaborative Group, the Childhood Arthritis and Rheumatology Research Alliance, and the American Society for Pediatric Nephrology

**Affiliations:** 1 Medical University of South Carolina, 135 Rutledge Ave, MSC 561, Charleston, SC 29425 USA; 2 The Children’s Hospital at Montefiore, 111 East 210th Street, Bronx, NY 10467 USA; 3 Stanford University School of Medicine, 291 Campus Drive, Stanford, CA 94305 USA; 4 Harvard Medical School, 300 Longwood Ave, Boston, MA 02115 USA; 5 Duke University, Box 3959, 2301 Erwin Road, Durham, NC 27710 USA; 6 Nationwide Children’s Hospital, 700 Children’s Drive, Columbus, OH 43221 USA; 7 Baylor College of Medicine, One Baylor Plaza, Houston, TX 77030 USA; 8 University of California San Francisco, 550 16th Street, 4th floor, San Francisco, CA 94158 USA

**Keywords:** Juvenile systemic lupus erythematosus, Refractory lupus nephritis, Renal flare, Therapy choices, Pediatric rheumatologist, Pediatric nephrologist

## Abstract

**Background:**

Consensus treatment plans have been developed for induction therapy of newly diagnosed proliferative lupus nephritis (LN) in childhood-onset systemic lupus erythematosus. However, patients who do not respond to initial therapy, or who develop renal flare after remission, warrant escalation of treatment. Our objective was to assess current practices of pediatric nephrologists and rheumatologists in North America in treatment of refractory proliferative LN and flare.

**Methods:**

Members of Childhood Arthritis and Rheumatology Research Alliance (CARRA) and the American Society for Pediatric Nephrology (ASPN) were surveyed in November 2015 to assess therapy choices (other than modifying steroid dosing) and level of agreement between rheumatologists and nephrologists for proliferative LN patients. Two cases were presented: (1) refractory disease after induction treatment with corticosteroid and cyclophosphamide (CYC) and (2) nephritis flare after initial response to treatment. Survey respondents chose treatments for three follow up scenarios for each case that varied by severity of presentation. Treatment options included CYC, mycophenolate mofetil (MMF), rituximab (RTX), and others, alone or in combination.

**Results:**

Seventy-six respondents from ASPN and foty-one respondents from CARRA represented approximately 15 % of the eligible members from each organization. Treatment choices between nephrologists and rheumatologists were highly variable and received greater than 50 % agreement for an individual treatment choice in only the following 2 of 6 follow up scenarios: 59 % of nephrologists, but only 38 % of rheumatologists, chose increasing dose of MMF in the case of LN refractory to induction therapy with proteinuria, hematuria, and improved serum creatinine. In a follow up scenario showing severe renal flare after achieving remission with induction therapy, 58 % of rheumatologists chose CYC and RTX combination therapy, whereas the top choice for nephrologists (43 %) was CYC alone. Rheumatologists in comparison to nephrologists chose more therapy options that contained RTX in all follow up scenarios except one (*p* < 0.05).

**Conclusions:**

Therapy choices for pediatric rheumatologists and nephrologists in the treatment of refractory LN or LN flare were highly variable with rheumatologists more often choosing rituximab. Further investigation is necessary to delineate the reasons behind this finding. This study highlights the importance of collaborative efforts in developing consensus treatment plans for pediatric LN.

**Supplementary Information:**

The online version contains supplementary material available at 10.1186/s12969-021-00627-w.

## Background

Systemic lupus erythematosus (SLE) is an autoimmune-mediated disease that can cause inflammation of multiple organ systems. The involvement of the kidneys, which occurs in over half of childhood SLE, significantly alters morbidity and mortality and therefore requires more aggressive immunosuppression [1, 2]. A meta-analysis showed 10-30 % higher prevalence of LN in childhood-onset SLE compared to adult-onset SLE [3], with up to 70 % of children with SLE having nephritis compared to about 50 % of adults with lupus [4, 5]. In the 1980 and 1990s, standard-of-care treatment with IV cyclophosphamide (CYC) was delineated by the National Institutes of Health for adults with proliferative LN [6–9]. More recently, mycophenolate mofetil (MMF) was found by the Aspreva Lupus Management Study investigators to be non-inferior to IV CYC [10], and both drugs are now used first-line for the treatment of proliferative LN. More black and Hispanic patients notably responded to MMF versus IV CYC [10].

While childhood-onset SLE comprises only about 15-20 % of all lupus patients [4, 5], it is difficult to perform large randomized controlled trials (RCTs) in pediatric LN given small numbers. The Childhood Arthritis and Rheumatology Research Alliance (CARRA) has developed consensus treatment plans (CTPs) in an effort to reduce heterogeneity in treatment and better enable future comparative effectiveness studies. CTPs for the induction treatment of proliferative LN were developed in 2012, and treatment arms include both IV CYC and MMF [11]. As in adults, current first-line treatment for initial diagnosis of proliferative LN encompasses CYC or MMF, along with the use of corticosteroids.

However, despite advances in the treatment of proliferative LN, a substantial number of patients never reach complete or even partial renal response after induction and are diagnosed as treatment refractory non-responders (NR). Eighteen to 69 % of adult patients with LN were resistant to CYC with variable definitions of treatment response [8, 12–15], whereas 5-48 % of patients treated with MMF were described as NR [15–17]. Likewise, at 6 and 12 months, 40-66 % and 65-75 % of children with proliferative LN achieved remission [18–22].

In the patients who do reach complete or partial renal response, unfortunately, renal flares can occur in about 50 % of adult-onset SLE after long term follow up [23, 24]. In children with SLE, renal flares can occur in 25-50 % of patients on therapy [25, 26]. Each subsequent flare leads to more kidney injury and heightened risk for kidney failure.

Patients who do not adequately respond to the CTP regimens, or who develop disease flare after remission, warrant change in treatment. Clinical guidance is lacking for this particularly vulnerable pediatric population, as there are no RCTs or CTPs for cases of NR/refractory LN. Several case series from single centers and case reports describe the use of other medications for treating refractory LN, including biologic agents that target B cell (rituximab, ofatumumab, belimumab) and T cell activity (abatacept, calcineurin inhibitors cyclosporine and tacrolimus) [27, 28]. Given the involvement of B cells in the pathogenesis of LN, rituximab (RTX) is one of the most widely employed rescue medications for patients who do not respond to standard treatment or in patients who flare [29].

Depending on the geographic location of a patient and availability of subspecialists in close proximity, pediatric nephrologists may be the primary subspecialists caring for patients with LN. Conversely, pediatric rheumatologists may be treating patients with LN as the leading organ system involvement in a SLE patient. Given this difficult-to-treat disease, treatment practices may vary by type of subspecialist, treating center, and even within practices.

In this study, members from both CARRA and the American Society for Pediatric Nephrology (ASPN) were surveyed to determine the current opinions and practices in the treatment of proliferative LN refractory to induction therapy and in patients who have a renal flare after achieving renal response in childhood-onset SLE.

## Methods

A web-based survey to assess immunosuppression treatment choices and level of agreement between pediatric rheumatologists and nephrologists for pediatric patients with proliferative LN was developed by the Pediatric Nephrology and Rheumatology Collaborative Group (PNR-CG) comprised of members of CARRA and the ASPN. Internal Review Board exemption for the study was obtained through Nationwide Children’s Hospital. The survey was sent to the membership of CARRA and the ASPN via Survey Monkey in November 2015. There were 76 respondents from ASPN and 41 respondents from CARRA representing approximately 15 % of the eligible members from each organization.

Two cases were presented in the survey: (1) Pediatric patient with proliferative LN refractory to 6 months of induction therapy with CYC (See Additional File 1) and (2) Pediatric patient with renal flare 3 months after achieving remission in response to induction therapy for proliferative LN (See Additional File 2).

Renal response and flare definitions defined in CARRA proliferative LN CTPs [11] were used in the cases. In summary, the core renal parameters were proteinuria, renal function, and urine sediment. Substantial response (complete remission) was defined as normalization of renal function, inactive urine sediment, plus spot protein/creatinine ratio < 0.2. Moderate and mild renal responses were defined as at least 50 % improvement (moderate) or 30–50 % improvement (mild) in two core renal parameters without clinically relevant worsening of the remaining core parameter. NR included any patient who did not qualify for mild, moderate, or substantial response. Proteinuric/nephrotic renal flare was defined as a persistent increase in urine protein/creatinine ratios to values > 0.5 after achieving complete response, or a doubling of proteinuria with values > 1.0 after achieving a partial response. Non-proteinuric/nephritic renal flare was defined as increase or recurrence of active urinary sediment with or without increase in proteinuria.

Physicians were surveyed on treatment choices for three fictional follow up scenarios for each case. The treatment choices included IV CYC, MMF, calcineurin inhibitors, RTX, belimumab, RTX in combination with IV CYC, and RTX in combination with MMF. Statistical analyses of differences between responses of pediatric nephrologists and rheumatologists were determined using Fisher’s Exact Test with *p* value < 0.05 (two-sided) considered statistically significant.

## Results

The survey was distributed to members of the ASPN and CARRA who completed fellowship programs in pediatric nephrology and pediatric rheumatology. The 76 respondents from ASPN and 41 respondents from CARRA represented approximately 15% of the eligible members from each organization. All respondents were board-eligible or board-certified in their subspecialty with the vast majority having at least 2 years of post-fellowship experience, and 45% of pediatric nephrologists and 60% of pediatric rheumatologists having greater than 10 years of experience. Fifty-two percent of pediatric nephrologists reported managing less than 25 patients with SLE, and half of pediatric rheumatologists reported managing 25 to 100 patients with SLE (Table 1). Only 51% of the pediatric nephrologists and 24% of the pediatric rheumatologists surveyed in this study follow a standard protocol for treatment of LN (Table 1).

**Table 1:** Demographics of survey respondents

	Pediatric nephrologists	Pediatric rheumatologists
**Total Respondents**	*N* = 76^a^	*N* = 41^a^
**Medical Centers**	*N* = 56	*N* = 15
**Peds Neph Board-Eligible**	100%	0%
**Peds Rheum Board-Eligible**	0%	100%
**Years in Practice**		
**< 2**	2%	7%
**2–5**	29%	17%
**6–10**	25%	17%
**> 10**	45%	60%
**# of Pediatric SLE Patients**		
**0–25**	52%	37%
**25–50**	14%	20%
**50–100**	19%	30%
**> 100**	6%	13%
**LN Standard Protocol**	51%	24%

^a^Approximately 15% of ASPN and CARRA membership

**Fig. 1:**
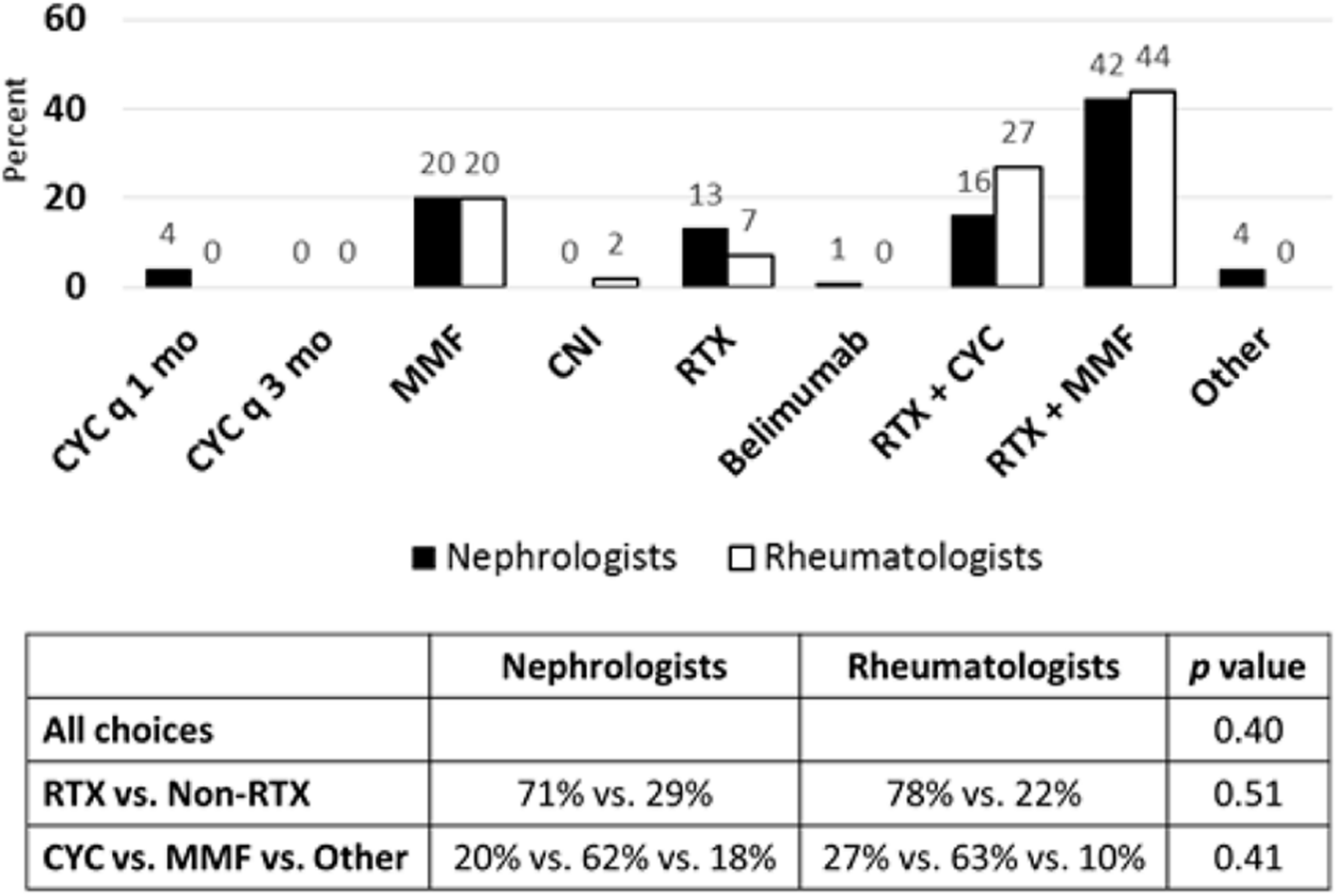
Treatment choices for LN refractory to induction therapy with renal insufficiency, nephrotic syndrome, and hematuria. Seventy-six nephrologists and forty-one rheumatologists responded to survey question. Treatment choices are depicted in graph. Statistical analysis of differences in responses between groups is depicted in chart. *P* value < 0.05 is considered statistically significant. (RTX = Rituximab, CNI = Calcineurin Inhibitor, MMF = Mycophenolate mofetil, CYC = Cyclophosphamide)

For the case of refractory class IV LN (See Additional File 1), a 16-year-old female failed induction therapy with 7 monthly doses of CYC 500–1000 mg/m^2^ IV in addition to steroids. Although fever, rash, and arthritis improved, a repeat renal biopsy showed persistent activity, little chronicity, and no evidence of membranous LN. In the first follow-up scenario (See Additional File 1), patient was found to have no renal response to induction therapy with hypertension, peripheral edema, hypocomplementemia, hypoalbuminemia, positive dsDNA antibody, persistent proteinuria, and active urine sediment, as well as a significant increase in serum creatinine. Survey respondents were asked to consider their next choice in immunosuppressive agent. Pediatric nephrologists and rheumatologists agreed with the top choice of MMF in combination with RTX (42 % vs. 44 %, Fig. 1). CYC in combination with RTX (16 % vs. 27 %, Fig. 1) and MMF alone (20 % vs. 20 %, Fig. 1) were the next most common choices for therapy. Overall, while the choices varied within groups, there was no difference between nephrologist and rheumatologist responses (*p* = 0.40).

**Fig. 2:**
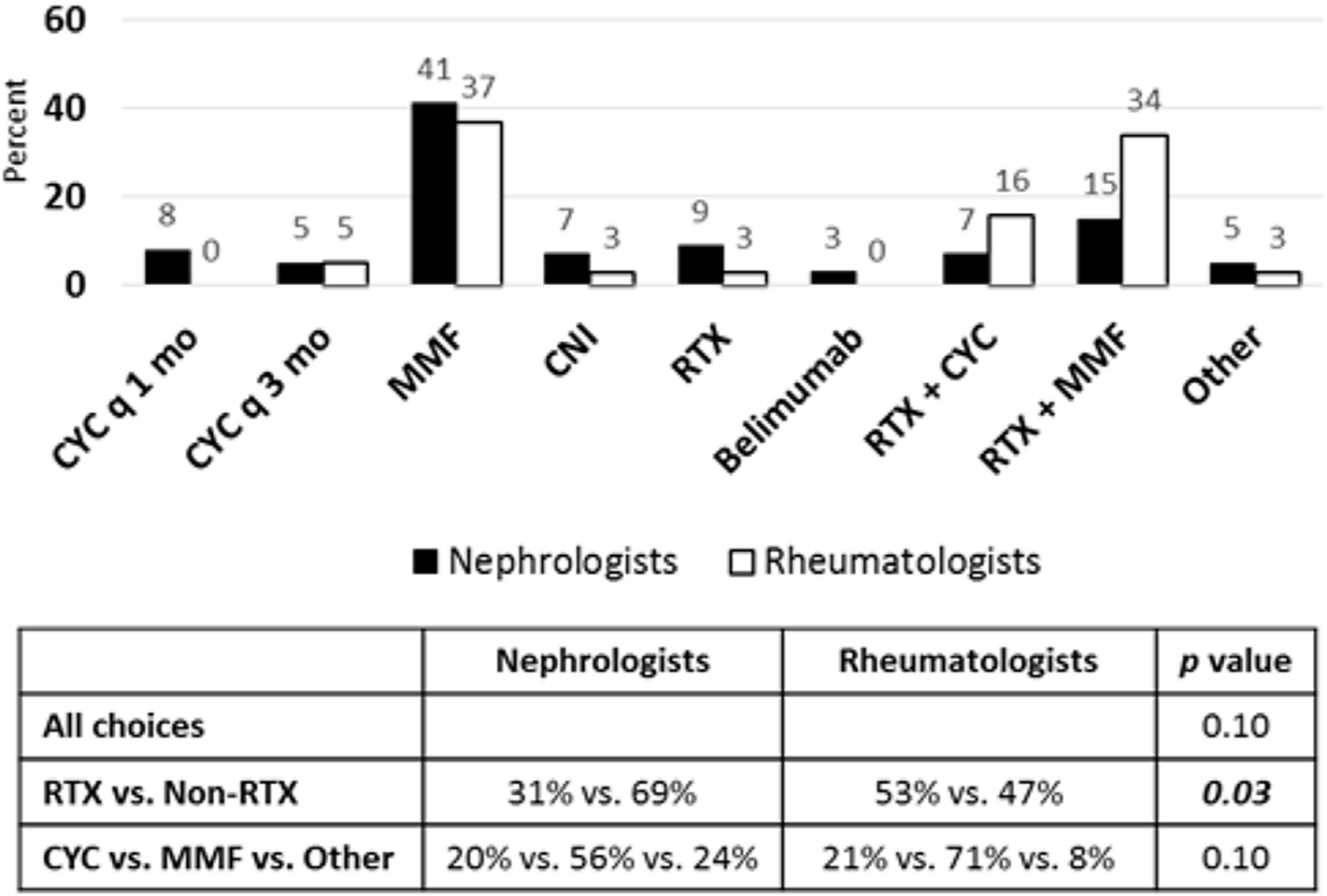
Treatment choices for LN refractory to induction therapy with nephrotic syndrome, improved hematuria and creatinine. Seventy-five nephrologists and thirty-eight rheumatologists responded to survey question. Treatment choices are depicted in graph. Statistical analysis of differences in responses between groups is depicted in chart. *P* value < 0.05 is considered statistically significant. (RTX = Rituximab, CNI = Calcineurin Inhibitor, MMF = Mycophenolate mofetil, CYC = Cyclophosphamide)

In the second follow-up scenario of refractory class IV LN after CYC induction therapy (See Additional File 1), the patient was found to have mild renal response with hypertension, peripheral edema, hypocomplementemia, hypoalbuminemia, positive dsDNA antibody, and persistent proteinuria, but improvement in serum creatinine and only mild hematuria. Pediatric nephrologists and rheumatologists chose MMF alone (41 % vs. 37 %, Fig. 2) and MMF in combination with RTX (15 % vs. 34 %, Fig. 2) as their top choices for next step in immunosuppressive therapy in this scenario. Overall, there was no difference between nephrologist and rheumatologist responses (*p* = 0.10). However, the majority of rheumatologists (53 %) chose therapies that involved RTX alone or in combination, compared to only 31 % of nephrologists that chose RTX therapies (Fig. 2). There was a difference between the groups of nephrologist and rheumatologist responses in choices of RTX-containing regimens versus choices without RTX (*p* = 0.03).

**Fig. 3:**
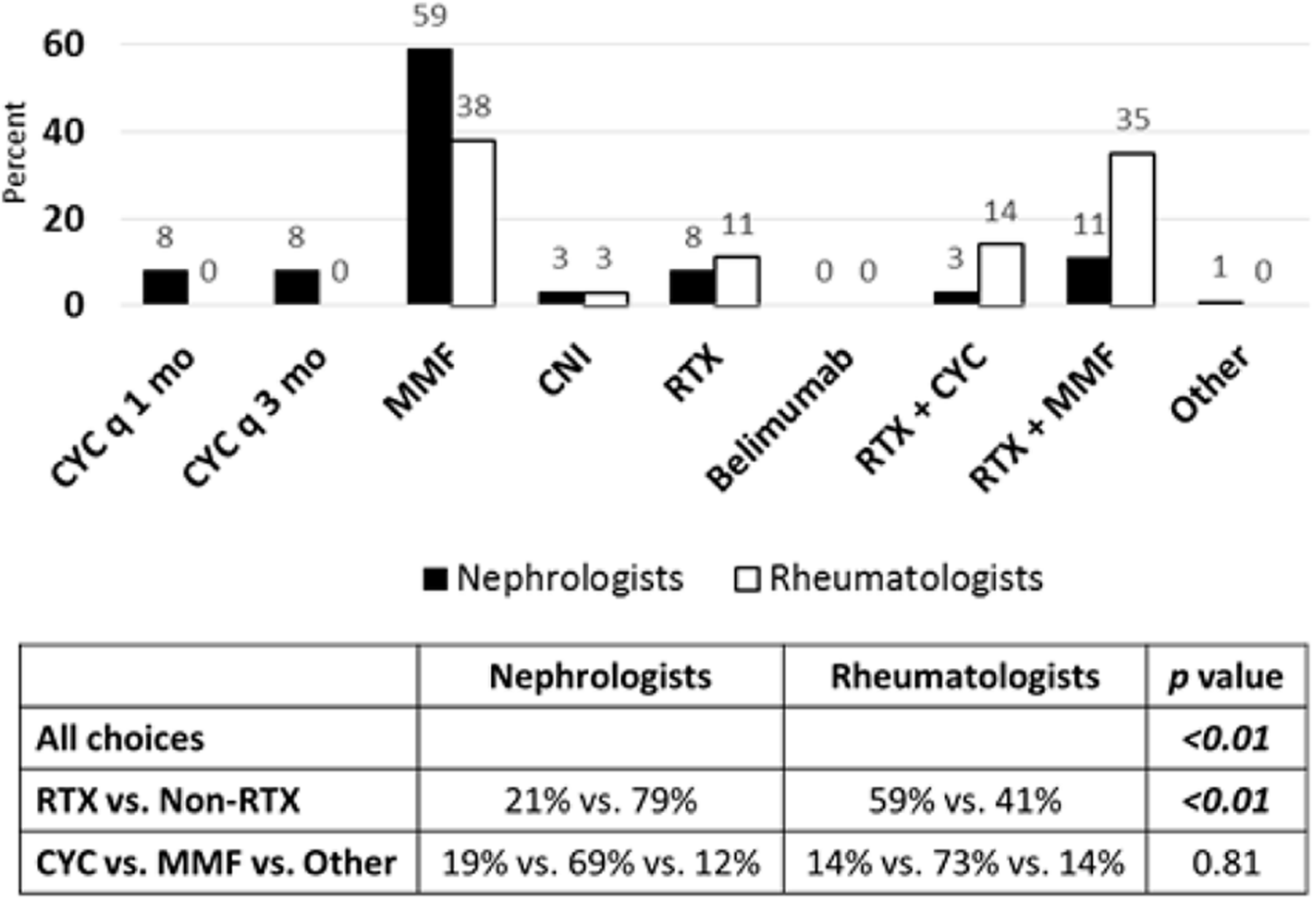
Treatment choices for LN refractory to induction therapy with hematuria, improved creatinine and proteinuria. Seventy-five nephrologists and thirty-seven rheumatologists responded to survey question. Treatment choices are depicted in graph. Statistical analysis of differences in responses between groups is depicted in table. *P* value < 0.05 is considered statistically significant. (RTX = Rituximab, CNI = Calcineurin Inhibitor, MMF = Mycophenolate mofetil, CYC = Cyclophosphamide)

In a third follow-up scenario of the refractory class IV LN case (See Additional File 1), the patient was found to have moderate renal response with persistent hypertension, resolution of peripheral edema, hypocomplementemia, hypoalbuminemia, positive dsDNA antibody, active urine sediment, but greater than 50 % improvement in serum creatinine and proteinuria. Whereas a majority of pediatric nephrologists chose MMF alone (59 %, Fig. 3), rheumatologists were split over use of MMF alone (38 %) or in combination with RTX (35 %) as their top choices for next step in immunosuppressive therapy. Overall, there was a difference between nephrologist and rheumatologist responses (*p* < 0.01), unlike in the prior follow-up scenarios. Additionally, rheumatologists again chose more therapies that involved RTX compared to nephrologists (59 % vs. 21 %, *p* < 0.01, Fig. 3). Thus, pediatric rheumatologists were more likely to use RTX in this scenario of active urine sediment but improvement in other renal parameters, and thus may be more aggressive with use of RTX. In all follow-up scenarios, there was no difference between groups of nephrologist and rheumatologist choices in therapies when considering additional CYC versus MMF or other options.

**Fig. 4:**
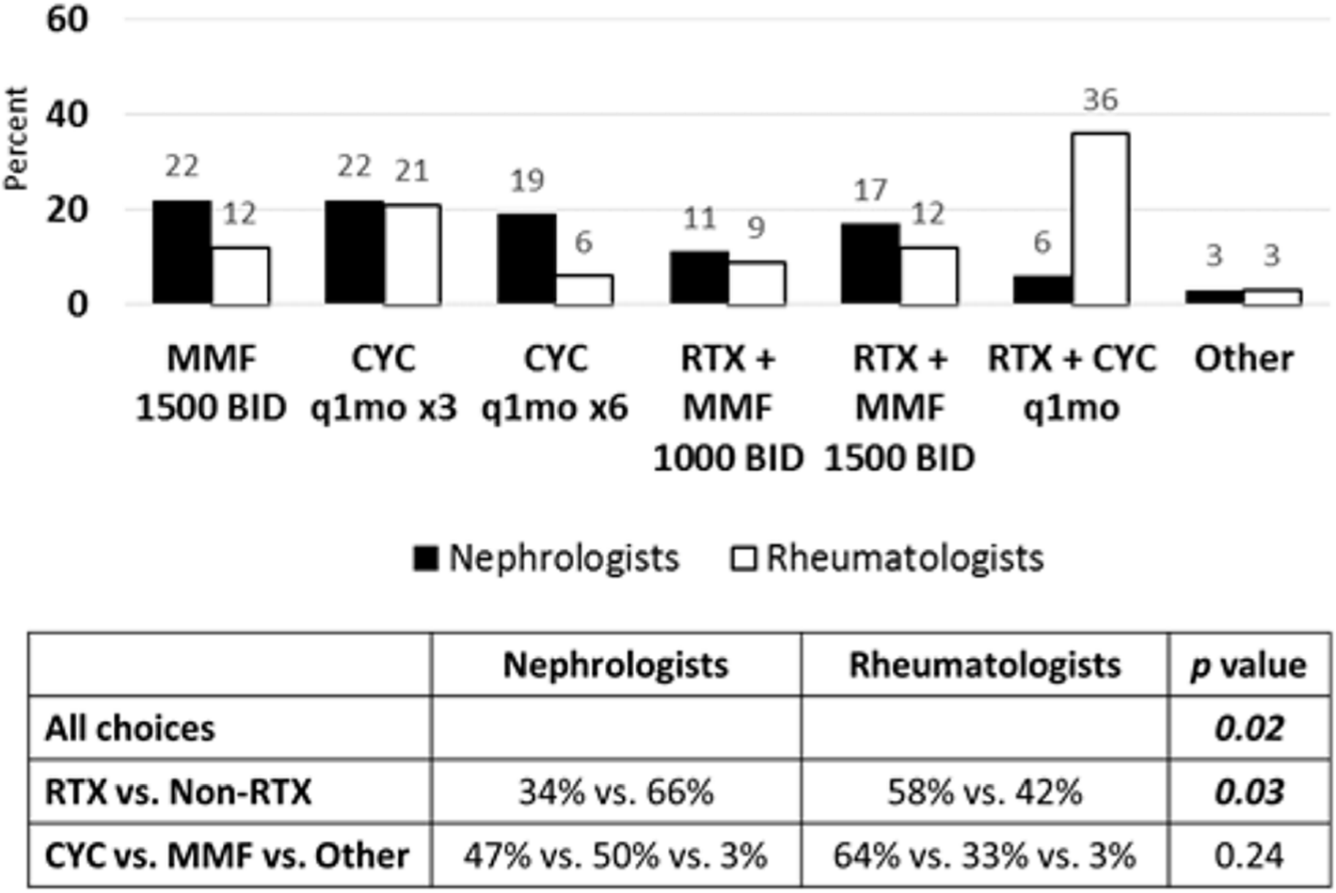
Treatment choices for renal flare in proliferative LN after achieving remission with CYC induction therapy. Sixty-four nephrologists and thirty-three rheumatologists responded to survey question. Treatment choices are depicted in graph. Statistical analysis of differences in responses between groups is depicted in chart. *P* value < 0.05 is considered statistically significant. (RTX = Rituximab, CNI = Calcineurin Inhibitor, MMF = Mycophenolate mofetil, CYC = Cyclophosphamide)

The second clinical vignette presented a case of a 12-year-old patient with SLE and class IV LN who achieved complete renal remission after induction therapy with CYC in addition to steroids for proliferative LN according to published CTP [11] (See Additional File 2). She was transitioned to MMF 1000 mg twice daily for maintenance therapy and tapering prednisone dose. Three months later, she developed nephrotic syndrome, hematuria with active urine sediment, hypocomplementemia, and high titer dsDNA antibody, without change in serum creatinine. She was diagnosed with renal flare, and repeat renal biopsy showed class IV LN with high activity and low chronicity scores. Pediatric nephrologists chose CYC alone (22 %) or increased dose of MMF to 1500 mg twice daily (22 %), whereas pediatric rheumatologists chose CYC in combination with RTX (36 %) or CYC alone (21 %) as their top choices for therapy (Fig. 4).

**Fig. 5:**
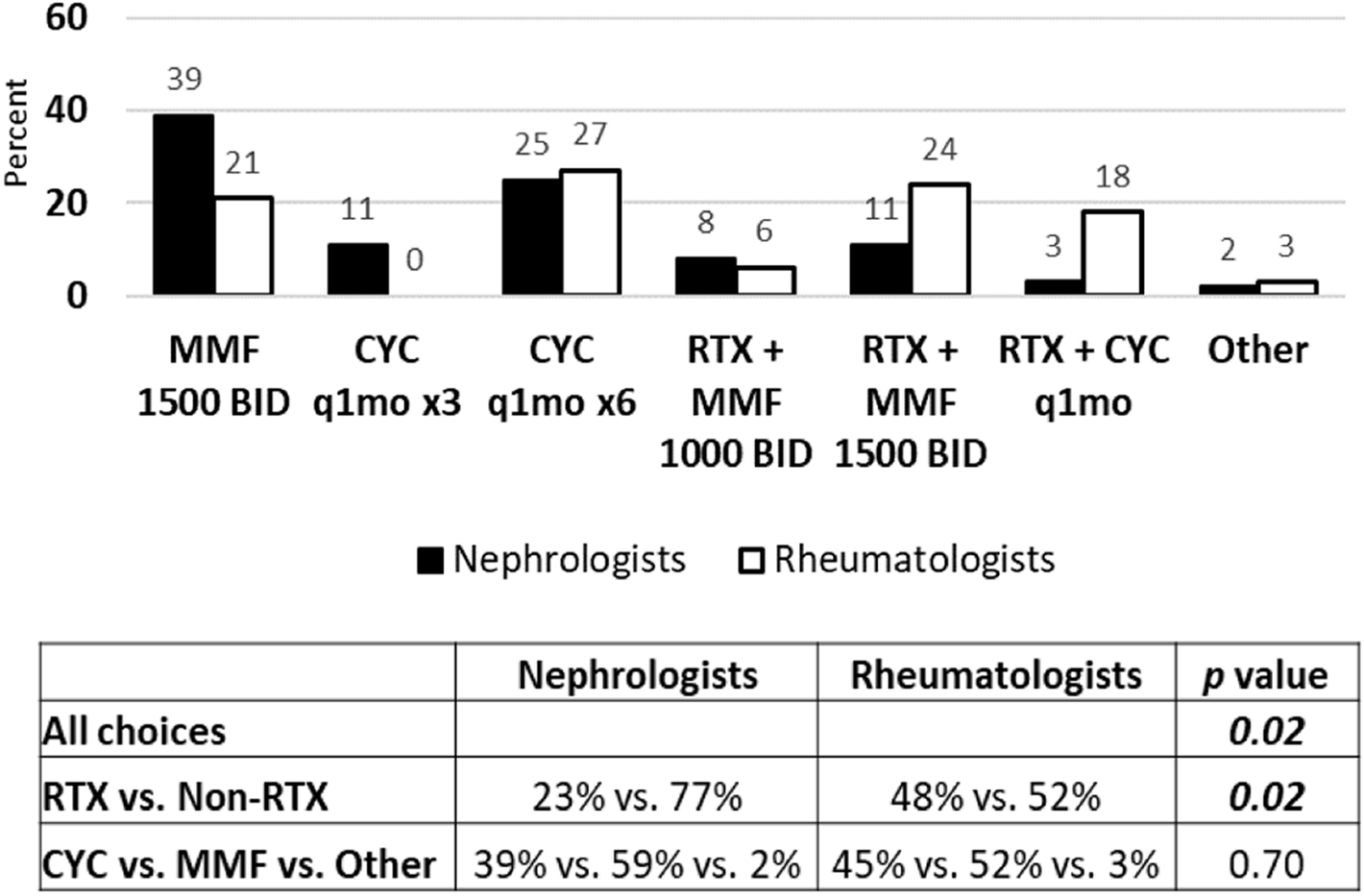
Treatment choices for renal flare in proliferative LN after achieving remission with MMF induction therapy. Sixty-one nephrologists and thirty-three rheumatologists responded to survey question. Treatment choices are depicted in graph. Statistical analysis of differences in responses between groups is depicted in table. *P* value < 0.05 is considered statistically significant. (RTX = Rituximab, CNI = Calcineurin Inhibitor, MMF = Mycophenolate mofetil, CYC = Cyclophosphamide)

If the second case used MMF as the induction agent instead of CYC at diagnosis (See Additional File 2), nephrologists chose increased dose of MMF to 1500 mg twice daily (39 %) or CYC alone (25 %) to treat renal flare (Fig. 5). Rheumatologists chose CYC alone (27 %) or MMF in combination with RTX (24 %) as their top choices for treatment of renal flare (Fig. 5).

**Fig. 6:**
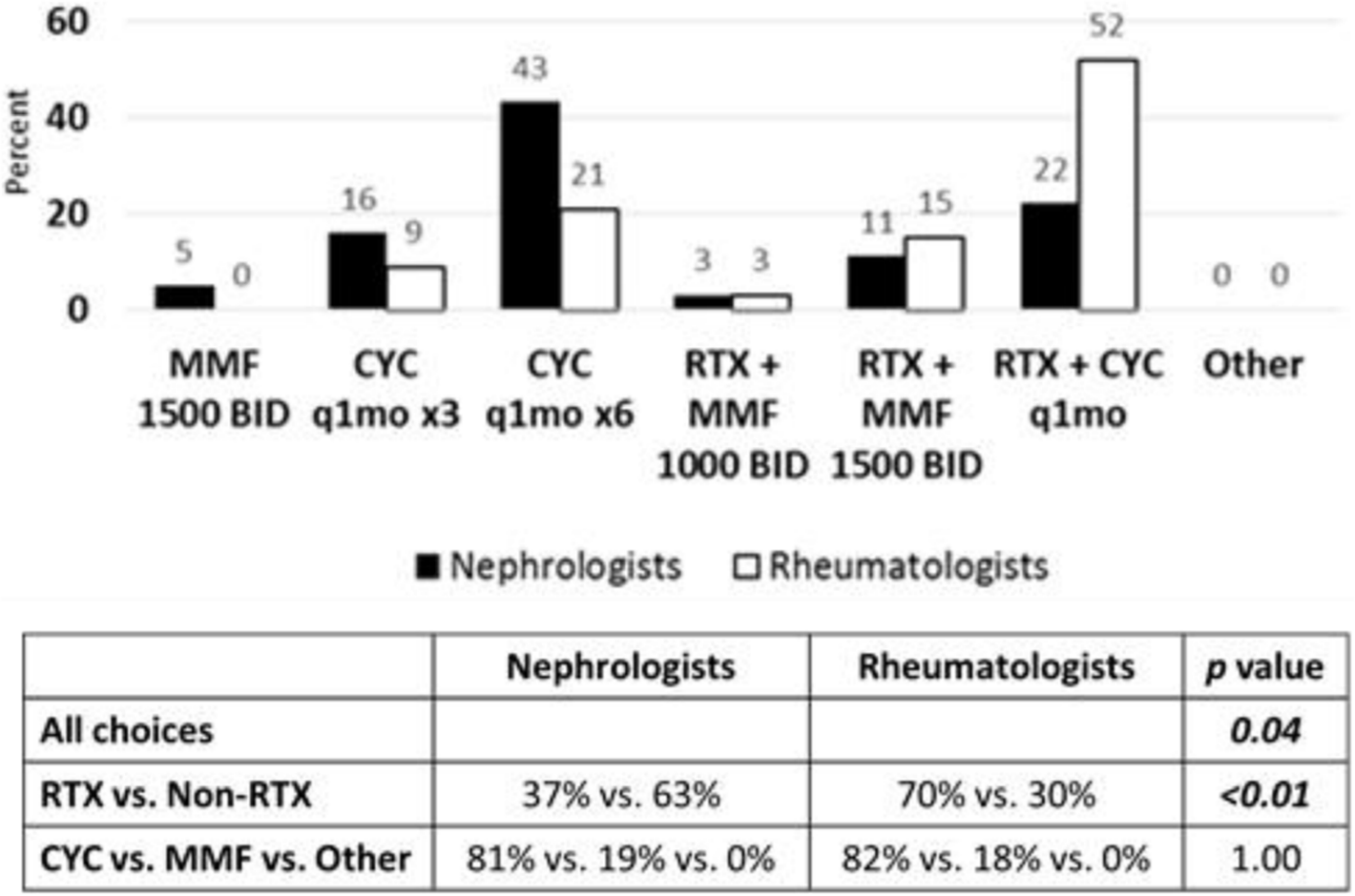
Treatment choices for renal flare after induction with renal insufficiency and rapidly progressive glomerulonephritis. Sixty-three nephrologists and thirty-three rheumatologists responded to survey question. Treatment choices are depicted in graph. Statistical analysis of differences in responses between groups is depicted in table. *P* value < 0.05 is considered statistically significant. (RTX = Rituximab, CNI = Calcineurin Inhibitor, MMF = Mycophenolate mofetil, CYC = Cyclophosphamide)

In the third follow up scenario of renal flare after complete renal remission was achieved with CYC and steroids followed by maintenance MMF therapy, the patient was also found to have elevated serum creatinine, rapidly progressive glomerulonephritis, and class IV LN on repeat renal biopsy (See Additional File 2). Nephrologists and rheumatologists (81 % vs. 82 %, Fig. 6) agreed that CYC with or without RTX were the best therapeutic choices for renal flare in this scenario, although more rheumatologists compared to nephrologists (52 % vs. 22 %, Fig. 6) would use CYC in combination with RTX.

In all three follow up scenarios involving case of renal flare after achieving remission with induction therapy, there were differences noted between nephrologists and rheumatologists (*p* = 0.02, 0.02, 0.04 respectively; Figs. 4, 5 and 6). Additionally, rheumatologists chose more therapies that involved RTX than nephrologists in all scenarios (*p* = 0.03, 0.02, < 0.01 respectively; Figs. 4, 5 and 6). There was no difference between groups of nephrologist and rheumatologist choices in therapies when considering additional CYC versus MMF or other options. Thus, pediatric rheumatologists chose RTX in more situations of renal flare than pediatric nephrologists, similar to the first case of proliferative LN refractory to induction therapy. Belimumab was included as a treatment option in both cases, however, was rarely chosen. Calcineurin inhibitors were also used in some patients by both rheumatology and nephrology and may have a role as well in resistant disease.

## Discussion

The approach to refractory proliferative LN after initial therapy with CYC or MMF is limited by the absence of evidence-based studies. This is particularly true in the case of pediatric LN where there is a lack of clinical trials. Thus, pediatric rheumatologists and nephrologists must make decisions on treatment of refractory LN based on retrospective cohort studies.

In this study, there are limitations regarding the definitions chosen for complete remission and flare. For instance, substantial response (complete remission) was defined as normalization of renal function, inactive urine sediment, plus spot protein/creatinine ratio < 0.2 consistent with the CARRA CTPs for the induction treatment of proliferative LN [11]. Recently, the adult LN community has changed the complete response definition to be far more liberal, with a spot protein/creatinine ratio < 0.5 and in more recent literature < 0.7 is now acceptable.

CTPs are a useful tool to compare effectiveness of currently practiced treatments in the clinical setting. The first step in developing CTPs for refractory proliferative LN in pediatric patients is to understand the clinical practices of pediatric rheumatologists and nephrologists.

This survey assessed the choices of immunosuppressive agents in clinical scenarios of refractory and relapsing childhood-onset proliferative LN. Treatment choices between nephrologists and rheumatologists were highly variable. Of note, pediatric rheumatologists and nephrologists did agree on the treatment of the most severe case of proliferative LN that was refractory to induction therapy, and both groups tended to choose more aggressive treatment options. However, there were differences in choice of therapies in the more moderate cases of refractory proliferative LN and in all cases of renal flare, particularly in the use of RTX.

While RTX in combination with MMF and corticosteroids did not meet primary endpoint of renal response in LN in patients 16–75 years of age after 1 year of treatment in the randomized, double-blind, placebo-controlled LUNAR clinical trial [30], small prospective studies of use of RTX alone or in combination with MMF for induction therapy of childhood LN have shown some efficacy and steroid-sparing effect [31, 32]. *Hogan et al.* looked at 12 patients with LN and found therapy with RTX + MMF combined with a rapid decrease in steroid appears to be an efficacious treatment for severe LN but was unfortunately associated with three varicella-zoster virus infections. *Basu et al.* assessed 44 patients with active LN and found that flare-free survival was significantly higher at 36 months with RTX compared with MMF and CYC (100 % for RTX vs. 83 % for MMF and 53 % for CYC, p = 0.006) [31]. Requirement of mean daily dosage of prednisone was significantly lower in RTX group at 36 months compared with other groups (RTX vs. MMF, *p* = 0.005; RTX vs. CYC, *p* = 0.0001) [32]. The degree of circulating B cell depletion by RTX appears to be critical for clinical effect and may explain the LUNAR trial results [33]. Meta-analyses of RTX treatment in refractory LN support an additional benefit [34, 35]. Moreover, several case series in childhood-onset LN report complete and partial remission at varying rates and steroid-sparing effect of RTX in refractory cases [29]. EULAR [36], ACR [37], and Kidney Disease Improving Global Outcomes (nonprofit organization developing and implementing evidence-based clinical practice guidelines) [38, 39] guidelines support the use of RTX as one of the treatment options in patients with refractory LN.

It is unclear as to why pediatric rheumatologists may treat more aggressively given rheumatologist’s increased choice of RTX in more moderate case scenarios of refractory and relapsing LN in this survey; however, it may be that more nephrologists reported using a standardized protocol. Unfortunately, this survey did not ask a question regarding which standardized protocol (KDIGO or adapt the ACR or EULAR guidelines for children). Pediatric rheumatologists may also be more comfortable with using RTX as this medication is also used for other rheumatologic indications with renal and non-renal manifestations; however, nephrologists also use rituximab for other nephrotic syndromes. This study highlights the importance of collaborative effort in developing CTPs for pediatric LN, as there are significant differences in the management of LN between pediatric rheumatologists and nephrologists.

Given the lack of FDA (Food and Drug Administration) approved therapies for LN and lack of guidelines for refractory LN, there are several biases and limitations of this case-based survey. Familiarity with treatment options with use for other indications may affect choice of therapy. Access to certain medications may be limited by insurance and thus affect physician experience with certain medications like belimumab. Belimumab was FDA approved for pediatric SLE in 2019 and recently has been shown as an effective add-on therapy for induction therapy for proliferative LN [40–42], but was not in widespread use at the time of this survey in 2015. The approach for nephrologists and rheumatologists is likely evolving now that the results of the BLISS-LN (Efficacy and Safety of Belimumab in Patients with Active LN) trial have been published [40]. Fifteen of pediatric nephrologists and rheumatologists in the ASPN and CARRA responded to the cases in the survey likely reflecting a sampling bias for those physicians who take care of more patients with SLE; although the majority of nephrologists surveyed manage less than 25 pediatric patients with LN. The low response rate to the survey is certainly a limitation of this study and may not be representative of the practice of these specialists in the United States and does not include any participants from Europe or other countries; however, the vast majority of pediatric rheumatologists and 49 % of pediatric nephrologists surveyed do not follow a standard protocol for treatment of LN and are not following CARRA CTPs. Thus, even with higher response rates, we would likely still see highly variable responses to the clinical scenarios presented. In the future, the study could be improved by sending repeat reminder emails to participants and by finding a way to incentivize participants who complete the survey. Another limitation to the study is the delay between the survey being sent to the participants (2015) and the publication. New drugs for SLE are not rapidly coming to market but it would be interesting to see if survey responses have changed in 2021 especially with Belimumab getting approved for the treatment of LN.

## Conclusions

Therapy choices for pediatric rheumatologists and nephrologists in the treatment of proliferative LN refractory to induction therapy or LN flare after remission were highly variable. In addition, there were differences between rheumatologists and nephrologists particularly in the use of RTX for the majority of scenarios presented in these cases. This provides an opportunity to work towards consensus to reduce heterogeneity in the treatment of refractory pediatric LN so that we can perform comparative effectiveness trials. This study highlights the importance of collaborative effort in developing CTPs for pediatric LN.

## Supplementary Information

**Additional file 1.**

**Additional file 2.**

## Data Availability

All data generated or analyzed during this study are included in this manuscript.

## Abbreviations

ACR: American College of Rheumatology
ASPN: American Society of Pediatric Nephrology
CARRA: Childhood Arthritis and Rheumatology Research Alliance
CTP: Consensus Treatment Plan
CYC: Cyclophosphamide
DNA: Deoxyribonucleic acid
EULAR: European League Against Rheumatism
FDA: Food and Drug Administration
ISN/RPS: International Society of Nephrology and the Renal Pathology Society
KDIGO: Kidney Disease Improving Global Outcomes
LN: Lupus nephritis
LUNAR: Lupus Nephritis Assessment with Rituximab study
MMF: Mycophenolate mofetil
NR: Non-responders
PNR-CG: Pediatric Nephrology and Rheumatology Collaborative Group
RCT: Randomized controlled trial
RTX: Rituximab
SLE: Systemic lupus erythematosus
