# Supplementary material for: Differences in rituximab use between pediatric rheumatologists and nephrologists for the treatment of refractory lupus nephritis and renal flare in childhood-onset SLE
Source: Pediatr Rheumatol Online J. 2021 Aug 30;19:137. doi: 10.1186/s12969-021-00627-w (PMC8404338; doi:10.1186/s12969-021-00627-w)
Supplement: Supplementary file 1 — Additional file 1. [file 12969_2021_627_MOESM1_ESM.pdf]

Refractory Proliferative Lupus Nephritis Case

A 16 year old female was diagnosed with SLE after presenting with fever, fatigue, hypertension (BP 160/95), palatal vasculitis, malar rash, arthritis, peripheral edema, cytopenias, hypocomplementemia, positive ANA and lupus serologies. Evaluation of renal involvement revealed serum creatinine 1.8, albumin 2.2, proteinuria, hematuria, pyuria, and rbc casts. Urine protein/creatinine (UPC) ratio was 3.1 and there was 4 grams of protein in 24 hour urine collection. Renal biopsy revealed diffuse proliferative glomerulonephritis with 50% crescents (and no chronic lesions), global and diffuse staining of all immunoglobulins and complements ("full house" staining), consistent with ISN/RPS class IV lupus nephritis. She underwent induction therapy with cyclophosphamide (CYC) 500-1000 mg/m^2 IV monthly for 6 months, in addition to IV pulse and oral steroid regimen, hydroxychloroquine, and lisinopril. A repeat biopsy was performed and showed 50% crescents, no chronicity, and no evidence of membranous lupus nephritis.

Given the 3 follow up scenarios below, what therapy (other than intensifying steroid and antihypertensive regimens) would you consider next in a patient with active nephritis and inadequate response to induction therapy?

1. Now 7 months into therapy, fever, rash, and arthritis have resolved. However, peripheral edema and hypertension (BP > 95th percentile on antihypertensive medications) persist. There is persistent hypocomplementemia and positive dsDNA titer. Serum creatinine has increased to 2.9, albumin is 2.4, UPC ratio is 3 with 60 rbc and 100 wbc/HPF and rbc casts in urine. Repeat 24 hour urine protein is 3 grams.
2. Now 7 months into therapy, fever, rash, and arthritis have resolved. However, peripheral edema and hypertension (BP > 95th percentile on antihypertensive medications) persist. There is persistent hypocomplementemia and positive dsDNA titer. Serum creatinine has decreased to 1.2, albumin is 2.1, UPC ratio is 3 with mild hematuria and no casts. Repeat 24 hour urine protein is 3.5 grams.
3. Now 7 months into therapy, fever, rash, arthritis, and peripheral edema have resolved. However, hypertension (BP > 95th percentile on antihypertensive medications) persists. There is persistent hypocomplementemia and positive dsDNA titer. Serum creatinine is 1.2, albumin 2.9, UPC ratio is 0.8 with 60 rbc and 100 wbc/HPF and rbc casts in urine. Repeat 24 hour urine protein is 1 gram.

Therapy options:

A) Continue IV CYC 500-1000 mg/m^2 monthly

B) Continue IV CYC 500-1000 mg/m^2 every 3 months

C) MMF

D) Calcineurin Inhibitors

E) Rituximab

F) Belimumab

G) Combination therapy- Rituximab with CYC

H) Combination therapy- Rituximab with MMF

I) Other
