# Supplementary material for: Differences in rituximab use between pediatric rheumatologists and nephrologists for the treatment of refractory lupus nephritis and renal flare in childhood-onset SLE
Source: Pediatr Rheumatol Online J. 2021 Aug 30;19:137. doi: 10.1186/s12969-021-00627-w (PMC8404338; doi:10.1186/s12969-021-00627-w)
Supplement: Supplementary file 2 — Additional file 2. [file 12969_2021_627_MOESM2_ESM.pdf]

Proliferative Lupus Nephritis Flare Case

12 year old female newly diagnosed with SLE is noted to have generalized edema with ascites, gross hematuria, and hypertension. Laboratory tests reveal hypocomplementemia, elevated dsDNA antibody level, azotemia, and urinary rbc and wbc casts. A renal biopsy reveals ISN/RPS Class IV (A-G) lupus nephritis. She receives induction therapy based on the published consensus treatment plan (CTP) (10). Other medications include hydroxychloroquine and lisinopril. One month after completing induction therapy, the urine is cell-free with a first-morning urine protein/creatinine (UPC) ratio of 0.1. Serum creatinine after induction is normal at 0.7 mg/dL (eGFR 100 mL/min/1.73m2) and all her serological abnormalities have resolved. Blood pressure is below the 90th percentile for age gender, and height, and she has no signs of extra-renal lupus manifestations. She is treated with mycophenolate mofetil (MMF) 1000 mg twice a day for consolidation/maintenance immunosuppression, while the prednisone dose is being weaned according to the CTP.

3 months later, she reports dark urine and swelling of her feet. Blood pressure is significantly above the 95th percentile for age, gender, and height. Urine studies show 3+ hematuria, 3+ proteinuria, granular and hyaline casts, in addition to numerous RBCs. UPC ratio on a first morning urine sample is 2.81, and serum albumin is 2.1g/dL. Serum creatinine is unchanged at 0.7 mg/dL (eGFR 100 mL/min/1.73m2). Additional laboratory tests reveal undetectable complement levels and high dsDNA antibody level. She has been compliant with all of her maintenance medications, and is currently on 7.5 mg of prednisone daily. You diagnose a renal flare, increase her prednisone dose, prescribe pulse steroids, and obtain a repeat biopsy, which, yet again, shows Class IV (A-G) lupus nephritis with high activity and low chronicity scores.

1. If the initial induction was with cyclophosphamide (CYC), which therapy option would you choose now?
2. If the initial induction was with oral MMF 1500 mg BID and subsequently lowered to 1000 mg BID for consolidation therapy/maintenance immunosuppression, which therapy option would you choose now?
3. Alternatively, the patient in the vignette above has an increase in the serum creatinine from 0.7 mg/dL (eGFR 100 mL/min/1.73m^2) to 3.1 mg/dL (eGFR 28 mL/min/1.73m^2) at her 3-month follow up visit after induction. You suspect rapidly progressive glomerulonephritis (RPGN). Repeat renal biopsy reveals Class IV (A/C-G) nephritis with 55% crescent formation in addition to significant activity and chronicity. What would be your next step in the management of this patient in addition to administering pulse steroids?

Therapy options:

A) Increase MMF dose to 1500 mg BID

B) Stop MMF and start monthly CYC infusions for 3 months

C) Stop MMF and start monthly CYC infusions for 6 months

D) Rituximab infusions combined with MMF at 1000 mg BID

E) Rituximab infusions combined with an *increased* MMF dose at 1500 mg BID

F) Stop MMF and give Rituximab infusions combined with monthly CYC infusions for 6 months

G) Other
